# Supplementary material for: Adolescent pregnancy in Mongolia: Evidence from Mongolia Social Indicator Sample Survey 2013–2018
Source: PLOS Glob Public Health. 2023 Apr 14;3(4):e0001821. doi: 10.1371/journal.pgph.0001821 (PMC10104280; doi:10.1371/journal.pgph.0001821)
Supplement: S1 Text — Table A: Characteristics of the sample, prevalence and 95% confidence Intervals, Mongolia SISS 2013. Table B: Characteristics of the sample, prevalence and 95% Confidence Intervals (CI), Mongolia SISS 2018. (DOCX) [file pgph.0001821.s001.docx]

**S1 Text**

**Table A:** Characteristics of the sample, prevalence and 95% confidence Intervals, Mongolia SISS 2013 (N*=1599).

| **Study variables** | **Unweighted Number**  **N (%)** | **Weighted**  **Number**  **N* (%)** | **Prevalence**  **(95%CI)** |
| --- | --- | --- | --- |
| **Community level factors** |  |  |  |
| *Type of residence (N=1599)* |  |  |  |
| Urban | 1080 (68.0) | 1133 (70.9) | 5.7 [4.3, 7.4] |
| Countryside | 509 (32.0) | 466 (29.1) | 8.1 [5.9, 10.9] |
| *Geographical zones (N=1599)* |  |  |  |
| Western | 239 (15.0) | 222 (13.9) | 1.6 [0.5, 4.8] |
| Khangai | 317 (19.9) | 300 (18.8) | 7.2 [4.8, 10.8] |
| Central | 212 (13.3) | 197 (12.3) | 11.1 [7.5, 16.1] |
| Eastern | 180 (11.3) | 103 (6.4) | 11.0 [6.9, 16.9] |
| Ulaanbaatar/Capital city | 641 (40.3) | 777 (48.5) | 5.6 [3.9, 7.9] |
| **Socio-demographic factors** |  |  |  |
| *Age (N=1599)* |  |  |  |
| 15-17 | 1125 (70.8) | 1116 (69.8) | 1.1 [0.6, 2.0] |
| 18-19 | 464 (29.2) | 483 (30.2) | 18.5 [15.1, 22.4] |
| *Education level (N=1599)* |  |  |  |
| No schooling | 36 (2.3) | 34 (2.1) | 12.1 [4.9,26.9] |
| Primary | 936 (58.9) | 911 (57.0) | 1.3 [0.7,2.2] |
| Secondary or more | 616 (38.8) | 654 (40.9) | 13.2 [10.5,16.3] |
| *Marital Status (N=1599)* |  |  |  |
| Currently unmarried | 1582 (99.6) | 1592 (99.6) | 6.1 [4.9, 7.5] |
| Formerly married | 7.0 (0.4) | 7.0 (0.4) | 73.4 [31.3, 94.3] |
| *Religion (N=1598)* |  |  |  |
| No religion | 689 (43.4) | 696 (43.5) | 8.8 [6.9, 11.3] |
| Buddha | 777 (48.9) | 782 (48.9) | 4.6 [3.3, 6.4] |
| Other | 122 (7.7) | 121 (7.6) | 3.5 [1.3, 9.5] |
| *Ethnicity (N=1594)* |  |  |  |
| Khalkha | 1217 (76.8) | 1246 (78.2) | 7.1 [5.7, 8.8] |
| Kazakh | 76 (4.8) | 71 (4.5) | 1.6 [0.2, 9.7] |
| Other | 291 (18.4) | 277 (17.4) | 3.9 [2.2, 6.9] |
| *Wealth index (N=1599)* |  |  |  |
| Poorest | 289 (18.2) | 256 (16.0) | 11.1 [7.8, 15.6] |
| Poor | 341 (21.5) | 331 (20.7) | 8.2 [5.6, 11.9] |
| Middle | 327 (20.6) | 323 (20.2) | 5.6 [3.6, 8.7] |
| Rich | 346 (21.8) | 367 (23.0) | 4.1 [2.4, 7.1] |
| Richest | 286 (18.0) | 321 (20.1) | 3.9 [2.2, 6.9] |
| **Individuals level factors** |  |  |  |
| *Ever smoked tobacco (N=1599*) |  |  |  |
| No | 1329 (83.6) | 1318 (82.5) | 5.2 [4.1, 6.7] |
| Yes | 260 (16.4) | 281 (17.5) | 11.7 [8.4, 16.1] |
| *Ever drunk alcohol (N=1599)* |  |  |  |
| No | 1053 (66.3) | 1028 (64.3) | 3.7 [2.7, 4.9] |
| Yes | 536 (33.7) | 571 (35.7) | 11.3 [8.8, 14.2] |
| *Combined tobacco and alcohol (N=1599)* |  |  |  |
| None | 983 (61.9) | 955 (59.7) | 3.5 [2.5, 4.8] |
| Only tobacco | 70 (4.4) | 73 (4.6) | 6.4 [2.6, 14.7] |
| Only alcohol | 346 (21.8) | 364 (22.7) | 9.9 [7.1, 13.6] |
| Both | 190 (12.0) | 207 (13.0) | 13.6 [9.5, 19.1] |
| **Media factors** |  |  |  |
| *Reading the newspaper (N=1599)* |  |  |  |
| Not at all | 278 (17.7) | 283 (17.8) | 9.5 [6.3,13.9] |
| Yes | 1296 (82.3) | 1303 (82.2) | 5.7 [4.5, 7.2] |
| *Listening to the radio (N=1599)* |  |  |  |
| Not at all | 1195 (75.2) | 1198 (74.9) | 6.2 [4.9, 7.8] |
| Yes | 394 (24.8) | 401 (25.1) | 6.8 [4.6, 9.8] |
| *Watching TV (N=1599)* |  |  |  |
| Not at all | 47 (3.0) | 44 (2.8) | 10.2 [4.2, 22.9] |
| Yes | 1542 (97.0) | 1555 (97.2) | 6.3 [5.1, 7.7] |
| *Ever used a computer (N=1599)* |  |  |  |
| No | 148 (9.3) | 135 (8.4) | 13.9 [9.3, 20.4] |
| Yes | 1441 (90.7) | 1464 (91.6) | 5.7 [4.5, 7.1] |
| *Ever used the internet (N=1599)* |  |  |  |
| No | 313 (19.7) | 291 (18.2) | 9.5 [6.7, 13.4] |
| Yes | 1276 (80.3) | 1308 (81.8) | 5.7 [4.4, 7.2] |
| *Has a mobile phone (N=1599)* |  |  |  |
| No | 61 (3.8) | 54 (3.4) | 5.5 [1.9, 15.1] |
| Yes | 1528 (96.2) | 1545 (96.6) | 6.4 [5.2, 7.8] |

N*= weighted number

**Table B:** Characteristics of the sample, prevalence and 95% Confidence Intervals (CI), Mongolia SISS 2018. (N*=1209).

| **Study variables** | **Unweighted Number**  **N (%)** | **Weighted**  **Number**  **N* (%)** | **Prevalence**  **(95%CI)** |
| --- | --- | --- | --- |
| **Community level factors** |  |  |  |
| *Type of residence (N=1219)* |  |  |  |
| Urban | 689 (56.5) | 877 (72.5) | 4.6 [3.1, 6.9] |
| Countryside | 530 (43.5) | 332 (27.5) | 6.1 [3.9, 9.5] |
| *Geographical zones (N=1219)* |  |  |  |
| Western | 350 (28.7) | 158 (13.0) | 2.1 [0.7, 6.3] |
| Khangai | 231 (18.9) | 188 (15.6) | 4.9 [2.6, 9.3] |
| Central | 142 (11.7) | 141 (11.7) | 7.5 [3.5, 15.2] |
| Eastern | 153 (12.6) | 75 (6.2) | 11.0 [6.8, 17.3] |
| Ulaanbaatar/Capital city | 343 (28.1) | 647 (53.5) | 4.5 [2.7, 7.5] |
| **Socio-demographic factors** |  |  |  |
| *Age in categories (N=1219)* |  |  |  |
| 15-17 | 915 (75.1) | 818 (67.7) | 1.2 [0.6, 2.5] |
| 18-19 | 304 (24.9) | 391 (32.3) | 12.9 [8.9, 18.3] |
| *Education level (N=1219)* |  |  |  |
| No schooling | 8 (0.7) | 8 (0.7) | 24.5 [3.7,73.1] |
| Primary | 872 (71.5) | 719 (59.5) | 1.0 [0.5,2.2] |
| Secondary or more | 339 (27.8) | 481 (39.8) | 10.6 [7.5,14.8] |
| *Marital Status (N=1219)* |  |  |  |
| Currently unmarried | 1216 (99.8) | 1207 (99.9) | 4.9 [3.5, 6.7] |
| Formerly married | 3.0 (0.3) | 2 (0.1) | *** |
| *Religion (N=1213)* |  |  |  |
| No religion | 438 (36.1) | 464 (38.6) | 5.3 [3.3, 8.2] |
| Buddha | 575 (47.4) | 613 (51) | 4.3 [2.5, 7.3] |
| Other | 200 (16.5) | 125 (10.4) | 7.6 [3.6, 15.4] |
| *Ethnicity (N=1217)* |  |  |  |
| Khalkha | 863 (70.9) | 945 (78.2) | 4.8 [3.3, 6.9] |
| Kazakh | 156 (12.8) | 58 (4.8) | 0.3 [0.03, 2.0] |
| Other | 198 (16.3) | 205 (17.0) | 7.4 [4.2, 12.7] |
| Wealth index (N=1219) |  |  |  |
| Poorest | 341 (28.0) | 209 (17.3) | 5.3 [3.2, 8.6] |
| Poor | 324 (26.6) | 231 (19.2) | 5.0 [2.7, 9.0] |
| Middle | 241 (19.8) | 237 (19.6) | 6.5 [3.3, 12.6] |
| Rich | 179 (14.7) | 272 (22.5) | 3.7 [1.5, 8.9] |
| Richest | 134 (11.0) | 260 (21.5) | 4.9 [2.1, 10.7] |
| **Individuals level factors** |  |  |  |
| *Ever smoked tobacco (N=1208)* |  |  |  |
| No | 1029 (84.7) | 953 (78.9) | 4.4 [2.9, 6.4] |
| Yes | 186 (15.3) | 254 (21.1) | 7.5 [4.1, 13.3] |
| *Ever drunk alcohol (N=1203)* |  |  |  |
| No | 979 (80.8) | 894 (74.4) | 3.3 [2.1, 4.9] |
| Yes | 233 (19.2) | 308 (25.6) | 10.2 [6.4, 15.9] |
| *Combined tobacco and alcohol (N=1202)* |  |  |  |
| None | 906 (75.0) | 820 (68.2) | 2.9 [1.9, 4.7] |
| Only tobacco | 69 (5.7) | 74 (6.1) | 6.4 [2.2, 17.3] |
| Only alcohol | 116 (9.6) | 128 (10.6) | 13.5 [7.0, 24.4] |
| Both | 117 (9.7) | 181 (15.0) | 7.9 [3.6, 16.4] |
| **Media factors** |  |  |  |
| *Reading the newspaper (N=1209)* |  |  |  |
| Not at all | 747 (61.3) | 768 (63.5) | 4.6 [3.0, 7.1] |
| Yes | 472 (38.7) | 441 (36.5) | 5.7 [3.5, 9.0] |
| *Listening to the radio (N=1209)* |  |  |  |
| Not at all | 958 (78.59) | 949 (78.5) | 5.4 [3.8, 7.7] |
| Yes | 261 (21.4) | 260 (21.5) | 3.5 [1.6, 7.3] |
| *Watching TV (N=1207)* |  |  |  |
| Not at all | 111 (9.1) | 89 (7.4) | 5.1 [1.8, 13.6] |
| Yes | 1105 (90.9) | 1117 (92.6) | 5.0 [3.6, 6.9] |
| *Ever used a computer (N=1205)* |  |  |  |
| No | 348 (28.6) | 265 (21.9) | 5.4 [3.0, 9.4] |
| Yes | 868 (71.4) | 940 (78.0) | 4.9 [3.3, 7.1] |
| *Ever used the internet (N=1209)* |  |  |  |
| No | 88 (7.2) | 62 (5.1) | 5.4 [1.8, 14.8] |
| Yes | 1131 (92.8) | 1147 (94.9) | 5.0 [3.6, 6.9] |
| *Has a mobile phone (N=1209)* |  |  |  |
| No | 87 (7.1) | 65 (5.4) | 5.4 [1.4, 18.7] |
| Yes | 1132 (92.9) | 1144 (94.6) | 5.0 [3.6, 6.8] |

N*= weighted number
