# Supplementary material for: Adolescent pregnancy in Mongolia: Evidence from Mongolia Social Indicator Sample Survey 2013–2018
Source: PLOS Glob Public Health. 2023 Apr 14;3(4):e0001821. doi: 10.1371/journal.pgph.0001821 (PMC10104280; doi:10.1371/journal.pgph.0001821)
Supplement: S2 Text — Table A: Unadjusted and adjusted odds ratios (OR) with 95%CI for factors associated with adolescent pregnancy in Mongolia, MSISS 2013. Table B: Unadjusted and adjusted odds ratios (OR) with 95%CI for factors associated with adolescent pregnancy in Mongolia, MSISS 2018. (DOCX) [file pgph.0001821.s002.docx]

**S2 Text**

**Table A:** Unadjusted and adjusted odds ratios (OR) with 95%CI for factors associated with adolescent pregnancy in Mongolia, MSISS 2013 (N=1588)

| **Study variable** | **Unadjusted Odd Ratio (UOR) [95%Cl]** | **P-value** | **Adjusted Odd Ratio (AOR) [95%Cl]** | **P-value** |
| --- | --- | --- | --- | --- |
| **Age in categories *(15-17 years, OR=1)*** |  |  |  |  |
| 18-19 years | 19.84 [10.65, 36.06] | <0.001 | 12.79 [6.06, 26.98] | <0.001 |
| **Religion *(No religion, OR=1)*** |  |  |  |  |
| Buddha | 1.99 [1.29, 3.07] | 0.002 | 1.88 [1.10, 3.20] | 0.021 |
| Other | 0.75 [0.25, 2.29] | 0.614 | 0.69 [0.27, 1.76] | 0.440 |
| **Wealth index *(Richest, OR=1)*** |  |  |  |  |
| Poorest | 1.05 [0.45,2.42] | 0.914 | 11.31 [4.50, 28.43] | <0.001 |
| Poor | 1.44 [0.67,3.08] | 0.35 | 3.85 [1.36, 10.90] | 0.011 |
| Middle | 2.17 [1.05, 4.40] | 0.037 | 3.98 [1.56, 10.18] | 0.004 |
| Rich | 3.03 [1.49, 6.18] | 0.002 | 1.10 [0.41, 2.95] | 0.850 |
| **Ever used contraceptive methods *(No, OR=1)*** |  |  |  |  |
| Yes | 26.58 [15.81, 44.67] | <0.001 | 14.44 [7.38, 28.26] | <0.001 |
| **Combined tobacco and alcohol (None, OR=1)** |  |  |  |  |
| Only tobacco | 1.89 [0.70, 5.14] | 0.207 | 3.03 [1.04, 8.79] | 0.042 |
| Only alcohol | 3.07 [1.94, 4.86] | <0.001 | 1.67 [0.84, 3.32] | 0.146 |
| Both | 4.42 [2.60, 7.49] | <0.001 | 1.81 [0.78, 4.21] | 0.167 |

**Table B:** Unadjusted and adjusted odds ratios (OR) with 95%CI for factors associated with adolescent pregnancy in Mongolia, MSISS 2018 (N=1206).

| **Study variable** | **Unadjusted Odd Ratio (UOR) [95%Cl]** | **P-value** | **Adjusted Odd Ratio (AOR) [95%Cl]** | **P-value** |
| --- | --- | --- | --- | --- |
| **Type of residence *(Urban, OR=1)*** |  |  |  |  |
| Countryside | 1.35 [0.71, 2.56] | 0.359 | 2.85 [1.17, 7.01] | 0.022 |
| **Age in categories *(15-17 years, OR=1)*** |  |  |  |  |
| 18 - 19 | 11.87 [5.33, 26.42] | <0.001 | 10.24 [4.34, 24.16] | <0.001 |
| **Ethnicity *(Khalkha, OR=1)*** |  |  |  |  |
| Kazakh | 0.05 [0.01, 0.42] | 0.005 | 0.09 [0.01, 0.71] | 0.022 |
| Other | 1.59 [0.76, 3.31] | 0.216 | 1.53 [0.59, 3.92] | 0.372 |
| **Ever used contraceptive methods *(No, OR=1)*** |  |  |  |  |
| Yes | 17.96 [8.65, 37.32] | <0.001 | 7.23 [2.83, 18.47] | <0.001 |
| **Combined tobacco and alcohol *(None, OR=1)*** |  |  |  |  |
| Only tobacco | 2.24 [0.67, 7.48] | 0.188 | 1.02 [0.17, 6.13] | 0.987 |
| Only alcohol | 5.07 [2.11, 12.19] | <0.001 | 3.15 [1.29, 7.64] | 0.011 |
| Both | 2.79 [1.08, 7.23] | 0.035 | 0.96 [0.26, 3.57] | 0.957 |
