# Supplementary material for: Adolescent pregnancy in Mongolia: Evidence from Mongolia Social Indicator Sample Survey 2013–2018
Source: PLOS Glob Public Health. 2023 Apr 14;3(4):e0001821. doi: 10.1371/journal.pgph.0001821 (PMC10104280; doi:10.1371/journal.pgph.0001821)
Supplement: Attachment — Submitted filename: Response to Reviewers.docx [file pgph.0001821.s003.docx]

A rebuttal letter

Dear reviewers,

Our manuscript had been previously submitted to PLOS ONE journal. However, we had to withdraw the manuscript because we did not receive any reviews for our submission as the editorial team was unable to find reviewers for our submission even after waiting several months. Hence, our decision to submit to another journal.

Regards,

The authors
