# Supplementary material for: Adolescent pregnancy in Mongolia: Evidence from Mongolia Social Indicator Sample Survey 2013–2018
Source: PLOS Glob Public Health. 2023 Apr 14;3(4):e0001821. doi: 10.1371/journal.pgph.0001821 (PMC10104280; doi:10.1371/journal.pgph.0001821)
Supplement: Attachment — Submitted filename: Response to Reviewers_PGPH .docx [file pgph.0001821.s004.docx]

**Response to Reviewers**

7 January 2023

**Manuscript ID: PGPH-D-22-00650**

Adolescent pregnancy in Mongolia: Evidence from Mongolia Social Indicator Sample Survey 2013-2018

**Authors: Bayaraa, Agho & Akombi-Inyang**

**Dear Editor-in-chief,**

Thank you for allowing us to revise and re-submit our manuscript. We are incredibly grateful for your insightful feedback that has strengthened our manuscript.

We have addressed all the comments raised by the reviewers in the revised version of the manuscript. Where we have a different opinion from the reviewers, we have clarified our position. We have submitted our revised manuscript with track changes as well as a clean version. We have also submitted supplementary tables in response to the reviewers’ comment which we believe will make our study stronger. Below, we provide a point-by-point response to the reviewers’ comments and, where possible, linked our responses to the appropriate sections in the revised manuscript. We hope that the revisions made have properly addressed the reviewers’ concerns and that our revised manuscript is accepted for publication in the journal.

**Reviewer #1**

Adolescent pregnancy is one of the important issues in obstetrics and gynecology. The authors have addressed about the adolescent pregnancy in Mongolia. I think the data will be interesting for both international and Mongolian readers.
There are few suggestions:

**Comment:** In table 1, the marital status of the weighted prevalence of adolescent pregnancy is 2.1 and 70.8 vs 95.3 and 4.8. It is suitable to explain why the numbers are so different.

**Response:** In table 1, 2.1 and 70.8 represent the weighted prevalence, while 95.3 and 4.8 represent the weighted number of the sample (n) which was expressed as a percentage (they add up to approximately 100). Prevalence and weighted number of the sample (n) are different and are calculated differently hence why they have different values. However, we have revised our study. The prevalence of marital status is now 5.6 and 78.7, while the weighted number of the sample (n) is now 99.7 and 0.3.

**Comment:** Age is grouped differently. Such as in table 1, 15-17,18-19 vs in table 2, it is <15, 16-17,18-19. What is the reason to change the age group?

**Response:** Thanks for bringing this to our attention. We have revised table 2 to read same as table 1 which is 15–17, 18-19.

**Reviewer #2**

**Comment:** The possibility of different interpretations arises from the fact that both individual and combined procedures will result in differing estimations. The feasibility of the unique technique should therefore be first examined by researchers, it is recommended. The pooled technique should only be used when it is logical to assume that the features and the domains of interest remain constant from cycle to cycle.

As a result, I suggest carrying out a statistical analysis for the 2013 dataset separately from the 2018 dataset. Before combining data to create pooled estimates, there are a few factors to consider. One could say that the considerations are an iterative process: analyze the rationale for why integrating is desired, ensure that integrating makes sense, integrate the data, compute estimates or conduct analysis, review the results, and determine if integrating still makes sense. Repeat. I won't be able to publish this if you don't give me adequate supporting scientific evidence.
-Please double-check and include the following before doing a pooled analysis, to be very specific. If you have previously completed this analysis, please provide a thorough explanation of the dataset merging process.

- When using pooled data to estimate the parameters in your regression analysis, keep an eye out for the impact of cycles.
- Are the 2013 and 2018 samples comparable? A five-year void exists. Because methods can change over time, the author should exercise caution.
-Which of the two sets of variables is most comparable? Are there any similarities between the two sets of estimates? Each variable should be checked independently, and the author should detail this in the paper's methods section. To determine if the two population parameters are related, the authors should do a formal hypothesis test on each set of estimations.

**Response:** we agreed with the reviewer, and we have reanalysed both the pooled and the 2013 and 2018 survey separately and results that are comparable between the three different analyses were reported in the manuscript and the estimate obtained from the 2013 and 2018 survey were reported as supplementary tables for the benefit of those readers interested in those results.

However, in a prevalence study, there is no hypothesis to test (see link below for details: <https://www.bmj.com/about-bmj/resources-readers/publications/statistics-square-one/13-study-design-and-choosing-statisti>

**Comment:** Please verify the following during the MLR analysis:
-According to the MLR, which has an OR of 70.1, those who live together or are married are more likely to be exposed to adolescent pregnancy. But I'm concerned that it doesn't adequately describe the whole thing. For instance, most research findings concur that married persons are more likely to have enduring relationships and more amenable to having children. Because this OR is so large and might be incorrect, please double-check your statistical analysis.

**Response:** The odd ratio is large due to the small sample size and has nothing to do with the analytical process. This was reflected in table 1, where about 133 vs 2675 for married/living together and never married, respectively was reported. This was also reflected in our separate analysis. However, after reanalysing the data as suggested, this variable was no longer significant.

**Comment:** Justify your choice to utilize the western region of Mongolia as the MLR's reference value. The western area of Mongolia has the lowest weighted prevalence of adolescent pregnancy, according to your data.

**Response:** We considered the western region of Mongolia as the reference value for easy epidemiological interpretation – meaning the western region has the lowest odds compared to others.
