# Supplementary material for: Adolescent pregnancy in Mongolia: Evidence from Mongolia Social Indicator Sample Survey 2013–2018
Source: PLOS Glob Public Health. 2023 Apr 14;3(4):e0001821. doi: 10.1371/journal.pgph.0001821 (PMC10104280; doi:10.1371/journal.pgph.0001821)
Supplement: Attachment — Submitted filename: RESPONSE TO REVIEWERS COMMENTS - 2.docx [file pgph.0001821.s005.docx]

**RESPONSE TO REVIEWERS’ COMMENTS**

**Editor’s comment:** Please also explain how you merged two distinct time periods in the study's procedure section and please consider the reviewer's comment about adding an additional study limitation related to this.

**Response: We have provided a brief description of how the merging was done (please see revised manuscript).**

**Comment:** I believe the author did not explain how she or he merged two distinct time periods in the study's procedure section, which is very important to include.

**Response:** **Thank you for your comment, we have now included a brief explanation of how the datasets were merged.**

**Comment:** In the BMJ article you provide, they address a frequentist statistical hypothesis rather than a scientific hypothesis. Various types of study are more exploratory and, hence, less hypothesis-driven. Between the two time points, the prevalence declined from 6.4% to 5%, hence it can be stated that the disease is less prevalent in 2018. But without statistical analysis, it is impossible to identify whether the numbers are identical or distinct. Prevalence may be a calculation you report as a result, but you can provide a general idea of it beforehand. I hypothesize, for instance, that the prevalence is +/- 1.4% than other part of the sample or that it is strongly correlated with socioeconomic characteristics. I refer to these scientific hypotheses. Therefore, you may include it in the study's limitation section.

**Response: Thank you for your comment, we have added the text below as one of the limitations of the study in the revised manuscript.**

*selection bias is inherent in combining two cross-sectional data sets of this nature because, in this study, participants selected in 2013 may be different from those participants selected in 2018 due to 5 years’ time lag.*
